# The use of video job-aids to improve the quality of seasonal malaria chemoprevention delivery

**DOI:** 10.1371/journal.pdig.0000165

**Published:** 2022-12-22

**Authors:** Susana Scott, Bienvenu Salim Camara, Michael Hill, Eugène Kaman Lama, Lansana Barry, Aurore Ogouyemi-Hounto, William Houndjo, Gauthier Tougri, Nombre Yacouba, Dorothy Achu, Marcellin Ateba, Mahamat Saleh Issakha Diar, Keziah L. Malm, Kofi Adomako, Paolo Djata, Wica Da Silva, Idrissa Cissé, Vincent Sanogo, Hadiza Jackou, Nnenna Ogbulafor, Bala M. Adu, Jamilu Nikau, Seynabou Gaye, Alioune Badara Gueye, Balla Kandeh, Olimatou Kolley, Tinah Atcha-Oubou, Tchassama Tchadjobo, Kovana Marcel Loua, Andre-Marie Tchouatieu, Ibrahima Mbaye, Maria-Angeles Lima-Parra, Abena Poku-Awuku, Jean Louis Ndiaye, Corinne Merle, Liz Thomas, Paul Milligan

**Affiliations:** 1 Faculty of Epidemiology and Population Health, London School of Hygiene and Tropical Medicine, London, United Kingdom; 2 Centre National de Formation et de Recherche en Santé Rurale de Maferinyah, Forécariah, Guinea; 3 Centre for Excellence in Learning and Teaching, London School of Hygiene and Tropical Medicine, London, United Kingdom; 4 National Control Malaria Programme, Ministry of Health, Conakry, Guinea; 5 National Malaria Control Programme, Ministry of Health, Cotonou, Benin; 6 National Malaria Control Programme, Ministry of Health, Ouagadougou, Burkina Faso; 7 National Malaria Control Programme, Ministry of Health, Yaoundé, Cameroon; 8 National Malaria Control Programme, Ministry of Health, N’Djamena, Chad; 9 National Malaria Control Programme, Ministry of Health, Accra, Ghana; 10 National Malaria Control Programme, Ministry of Health, Bissau, Guinea Bissau; 11 National Malaria Control Programme, Ministry of Health, Bamako, Mali; 12 National Malaria Control Programme, Ministry of Health, Niamey, Niger; 13 National Malaria Elimination Programme, Ministry of Health, Abuja, Nigeria; 14 National Malaria Control Programme, Ministry of Health, Dakar, Senegal; 15 National Malaria Control Programme, Ministry of Health, Banjul, The Gambia; 16 National Malaria Control Programme, Ministry of Health, Lomé, Togo; 17 University of Gamal Abdel Nasser in Conakry, Conakry, Guinea; 18 MMV Medicines for Malaria Venture, Geneva, Switzerland; 19 Université de Thiès, Thiès, Senegal; 20 La Cooperativa Humanitaria, Girona, Spain; 21 UNDP/UNICEF/World Bank/WHO Special Programme for Research and Training in Tropical Diseases (TDR)/ World Health Organization (WHO), Geneva, Switzerland; 22 University of York, York, United Kingdom; University of British Columbia, CANADA

## Abstract

Mobile phones are increasingly used in community health programmes, but the use of video job-aids that can be displayed on smart phones has not been widely exploited. We investigated the use of video job-aids to support the delivery of seasonal malaria chemoprevention (SMC) in countries in West and Central Africa. The study was prompted by the need for training tools that could be used in a socially distanced manner during the COVID-19 pandemic. Animated videos were developed in English, French, Portuguese, Fula and Hausa, illustrating key steps for administering SMC safely, including wearing masks, washing hands, and social distancing. Through a consultative process with the national malaria programmes of countries using SMC, successive versions of the script and videos were reviewed to ensure accurate and relevant content. Online workshops were held with programme managers to plan how to use the videos in SMC staff training and supervision, and the use of the videos was evaluated in Guinea through focus groups and in-depth interviews with drug distributors and other staff involved in SMC delivery and through direct observations of SMC administration. Programme managers found the videos useful as they reinforce messages, can be viewed at any time and repeatedly, and when used during training sessions, provide a focus of discussion and support for trainers and help retain messages. Managers requested that local specificities of SMC delivery in their setting be included in tailored versions of the video for their country, and videos were required to be narrated in a variety of local languages. In Guinea, SMC drug distributors found the video covered the all the essential steps and found the video easy to understand. However, not all key messages were followed as some of the safety measures, social distancing and wearing masks, were perceived by some as creating mistrust amongst communities. Video job-aids can potentially provide an efficient means of reaching large numbers of drug distributors with guidance for safe and effective distribution of SMC. Not all distributors use android phones, but SMC programmes are increasingly providing drug distributors with android devices to track delivery, and personal ownership of smartphones in sub-Saharan Africa is growing. The use of video job-aids for community health workers to improve the quality delivery of SMC, or of other primary health care interventions, should be more widely evaluated.

## Author summary

During the COVID-19 pandemic, it was important to ensure that core malaria prevention strategies continued. Seasonal malaria chemoprevention (SMC) is the monthly administration of chemoprevention drugs to children to prevent malaria in areas with highly seasonal malaria transmission. In partnership with 13 National Malaria Control Programmes in West and Central Africa, we developed a user-friendly training video to support safe delivery of SMC during COVID-19. The use of the video was evaluated through a case study in Guinea and an online workshop with the programme coordinators. SMC drug distributors found the video covered all the essential steps and was easy to understand. However, not all key messages were followed as social distancing and wearing masks were perceived by some as creating mistrust. Programme coordinators found the video useful as it reinforces messages and can be viewed many times. Video content needed to be adapted to the local setting and narrated in local languages. Not all distributors use android phones but many SMC programmes now provide drug distributors with android devices to use during SMC delivery, so that video job-aids could be more widely used.

## Introduction

Community health worker (CHW) programmes rely on effective training and supervision [1]. The increasing use of mobile devices by community health programmes in Africa [2], and increasing personal ownership of smartphones [3], creates opportunities to use video to explain complex tasks during pre-service training and for community health workers to refer to as job-aids. However, although mobile phones have been used in primary health programmes [4,5], the use of video job-aids has not yet been widely exploited or evaluated in this context. We investigated the role of video job aids to improve the quality of Seasonal Malaria Chemoprevention (SMC) delivery. The study was prompted by the need for training tools that could be used in a socially-distanced manner during the COVID-19 pandemic, and which would familiarise distributors with modified procedures for drug administration adopted to minimise the risk of spreading COVID-19.

### Delivery of seasonal malaria chemoprevention

SMC involves intermittent administration of the antimalarial drugs sulfadoxine-pyrimethamine and amodiaquine (SPAQ) to children to prevent malaria. It is used in areas with highly seasonal malaria transmission in West and Central Africa where the majority of malaria cases, especially severe cases, occur in young children during and shortly after the rainy season [6]. SMC involves contacts with the health system every 4 weeks, for up to 5 months each year, generally through door-to-door campaigns. Drug distributors explain the purpose of SMC; check eligibility of children in the household; refer any who are unwell to the nearest health facility or community health worker so that they can be assessed and treated appropriately for their illness; and administer to the other eligible children the first dose of the 3-day regimen, leaving the blister pack with the caregiver with instructions to administer the remaining two doses, one on each of the next two days. They also explain the adverse reactions to look for, and that care should be sought if the child is unwell after treatment. In some countries, distributors or other community volunteers return on the second and third day to ensure that all the doses are administered and to treat any eligible children who had been missed in earlier visits. Treatments are recorded on a record card retained by the caregiver, and on tally sheets and in a register, however increasingly countries are replacing paper records with electronic recording on android devices [2].

SMC campaigns require substantial human resources. For example, the ACCESS-SMC project in 2016 used about 60,000 people (drug distributors, supervisors, and health facility workers) to treat about 7million children in seven countries [7]. SMC programmes have been implemented in 13 countries reaching over 35 million in 2021. The training and supervision to ensure quality of SMC delivery on this scale is a massive undertaking. Training is usually organised using a cascade approach beginning with national training of trainers followed by cascading down to regional, district and facility level [7]. Training usually occurs immediately before the SMC campaign, and during the campaigns supervisors are employed to oversee and manage the drug distributors.

### Modifications for safe delivery of SMC during the COVID-19 pandemic

As the COVID-19 pandemic unfolded, WHO highlighted the importance of ensuring continuity of essential services that can be delivered safely at community level [8]and produced specific guidelines for sustaining SMC delivery [9], with modifications to ensure training and delivery could be undertaken safely. Recommendations included adapting training to maintain social distance, mask-wearing, hand washing, and asking the caregiver to administer the medication under observation (rather than the drug being administered by the distributor). The guidelines suggested the use of innovative training methods such as videos, text messages or apps, providing training materials digitally to distributors and supervisors, and encouraging them to work on training in their own time [9].

This paper describes the development and evaluation of video job-aids to support the safe delivery of SMC in the context of COVID-19, and to improve the quality of SMC delivery.

## Materials and methods

### Scope and purpose of the video job-aids

The purpose of the video was to show drug distributors the steps involved in SMC administration, to be viewed by distributors during their work as a job-aid. These steps are detailed in Table 1. The video content was limited to the household visit and did not attempt to describe other activities of SMC campaigns such as planning, supervision, community publicity, stock reconciliation etc, as the target audience was the SMC drug distributors. However, we anticipated that the video would also be useful also as a resource for trainers and supervisors, and might be viewed by caregivers, and by others wanting to understand the purpose of SMC and how SMC is delivered.

**Table 1:** Steps involved in SMC delivery, and adaptions made in the context of COVID-19, described in the video job-aid.

Steps to be followed during door-to-door visits	COVID-19 adaptations
**1. Arrive at the household**, introduce yourself and explain the purpose of SMC	- Put on a mask either before you arrive or just as you arrive at the household. - Wash your hands before your visit, either before or at the start of the visit - Make sure you keep a safe distance from the caregivers and children at all times.
**2. Check eligibility**:	
*Aged at least 3 months*, and was under 5 years old at the time of the first cycle	
*Signs of illness*: children who have a fever or are otherwise unwell, should be referred to the nearest health worker to be tested for malaria, so that they can received artemisinin combination treatment if they have malaria; they may receive SMC if they test negative.	
*Allergies to SMC drugs*: Children with known history of allergy to SMC drugs or to other sulfa-containing medicines should not receive SMC	
*Other medicines*: children who have taken a dose of SP or AQ in the past 4 weeks, and children who are currently taking other sulfa-containing drugs (e.g. cotrimoxazole), should not receive SMC	
**3. Select the correct SMC blister pack** for the child’s age, and administer the dose of SP and the first dose of AQ	Select the correct SMC blister pack. Place in front and step back. Ask the caregiver to give the first dose of SP and the first dose of AQ to the eligible child. Explain to the caregiver how to administer the drugs slowly with a small amount of water.
**4. Observe**, and repeat the doses if the child vomits all of the medication	Tell the caregiver if the child vomits ALL the medicine in the next 30 minutes, they can be re-dosed.
**5. Record the date on the child’s SMC card**, and record the treatment on a tally sheet and in the register, or electronically	When you have completed the SMC record card, place it down and step back, so the caregiver can take it.
**6. Explain to the caregiver** the importance of completing the course of tablets by administering a dose of AQ on each of the next two days, to keep the tablets in a safe place, and not to use the tablets to treat anyone else	
**7. Explain that side effects can occur** and to take the child to a health worker if they become unwell after treatment	
**8. Explain that SMC does not give complete protection**, the child should sleep under a LLIN, and the child should be taken to a health worker promptly if they have a fever	
**9. Remind the caregiver of the date of the next SMC cycle**	Tell the caregiver to wash their hands once you have left the household.

It was considered important that the messages should be clear from the animation without relying on the narration for explanation, and we aimed to keep the video to no more than 5 to 6 minutes in length, and with a file size small enough that could be easily and quickly transmitted via phone apps such as WhatsApp or Bluetooth.

Messages should be consistent with current guidelines as detailed in the WHO SMC Field Guide [6] and SMC training manuals and job-aids (such as those used for the ACCESS-SMC project, [7]. Animation was chosen so that the video would look more generic compared to live-action, as well as being a solution to the challenges of filming live action due to COVID-19 travel restrictions.

### Production process

The video job-aid was developed in three stages: pre-production, production, and post-production (Table A in S1 File). An advisory group including the thirteen national malaria control programmes involved in SMC, were consulted throughout the production process. This group reviewed the initial script in English or French, but found it easier to comment on accuracy and suitability of content when they were sent the early versions of the animation to view alongside the scripts.

### Content versions and languages

National programmes requested local specificities of SMC delivery be included in tailored versions of the video for their country. This had to be balanced with the aim that the videos should promote consensus good practice in a standardized manner. For example, in Guinea Bissau and Benin, drug distributors mixed the tablets with water in a large spoon, as opposed to in a cup, to ensure only a small amount of water can be added, this was included in the videos. Most countries trained distributors to administer the dose of SP and AQ together first, but in some instances the training was to administer the two tablets separately, this was felt to be unnecessary and was not included in the videos. As far as possible, local variations were incorporated within one video to avoid having multiple versions. In most SMC programmes the first daily dose is administered by the distributor (or by the caregiver under observation by the distributor), the remaining doses left with the caregiver to administer on the next two days. Some countries require distributors to visit over three days to supervise administration of each of the three daily doses, but this was not included in the videos as during the COVID-19 pandemic this practice was discouraged by WHO to minimise risks of COVID transmission [9].

In the Gambia, Ghana and Benin, mobile devices were used to record treatments rather than tally sheets and registers for the 2020 campaigns. This digitalisation was expanded in the 2021 campaigns to parts of Nigeria, Burkina Faso and Guinea. Thus, the videos were revised for the 2021 campaigns to reflect both paper and digital recording of SMC delivery.

Increasingly, other interventions are combined with SMC such as nutritional screening and referral of severely malnourished children, these activities were not included in the videos but it may be useful to add them in future, or cover them in separate videos.

The country-specific adaptions included in the SMC video job-aid are detailed in Table B in S1 File.

Narrations in a range of local languages were requested. A final script (S2 File) and animation was developed in both French and English. It was then then translated into Portuguese (for Guinea Bissau) and Hausa (primarily for Nigeria and Niger), and Fula, (S2 File) and the translations independently verified for accuracy before narration audios were recorded. To keep the number of versions of the animation manageable, the animation text where it appeared in the video was limited to English, French or Portuguese versions.

For four countries (Mali, Guinea, Benin and Togo), the NMCP coordinator recorded an introduction which was included at the start of the video, to endorse and promote its use.

### Dissemination of the videos

**Fig 1:**
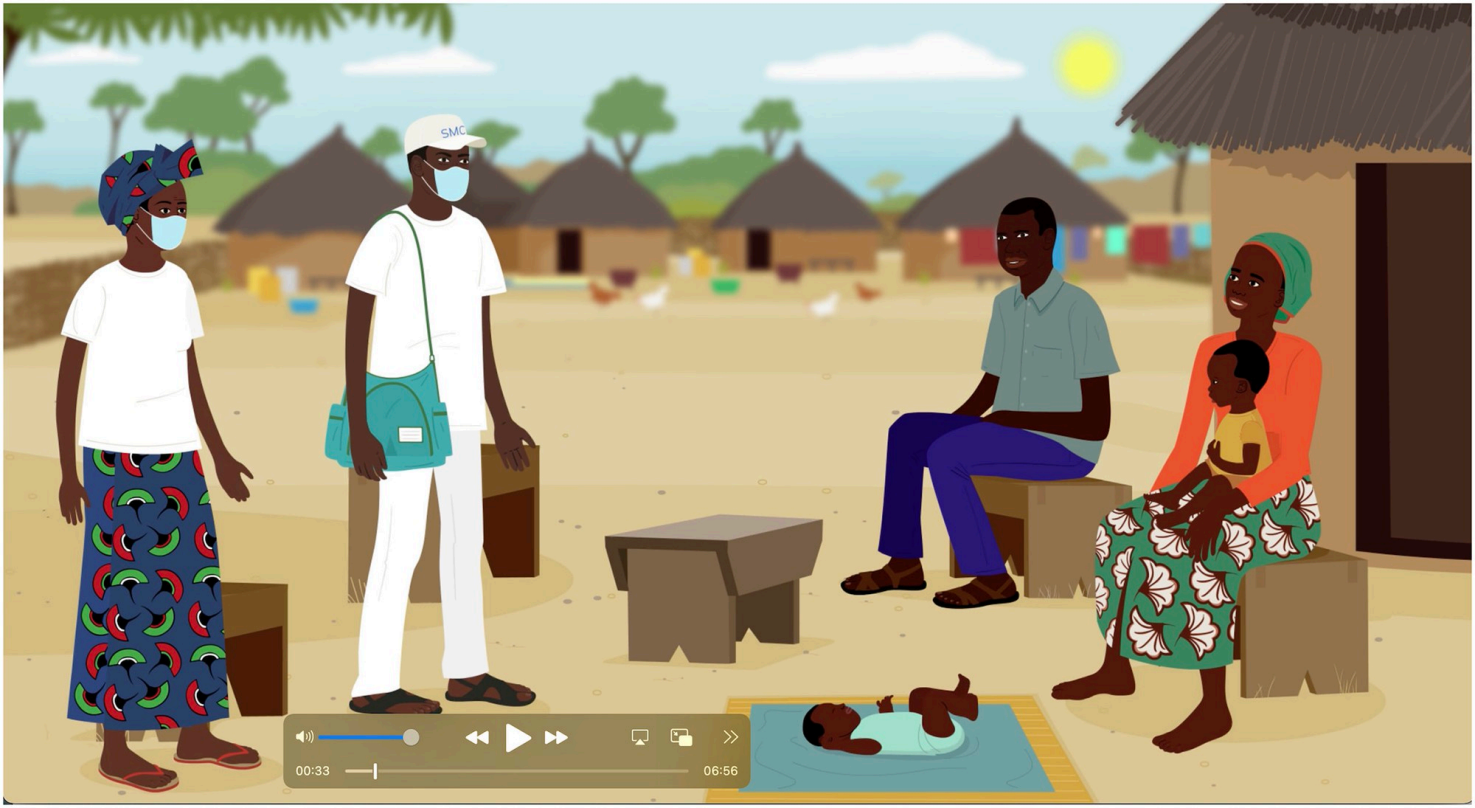
Screenshot from the video job-aid.

The videos were shared via WhatsApp to all national coordinators. They were then asked to distribute the video to their NMCP teams and to cascade it down to their SMC teams. The videos were also made available for downloading with varying resolutions on the LSHTM website, the TDR/WHO YouTube channel and the SMC Alliance website (Fig 1)

The video was developed during the 2020 SMC campaigns and final versions circulated after the start of the campaigns. Six countries (Nigeria, The Gambia, Guinea, Mali, Togo and Chad) used the videos. The video was shared via WhatsApp to the national coordinators who then cascaded the video down to the local SMC team. In The Gambia, the video was uploaded on the handheld tablet PCs that drug distributors used to record SMC delivery, allowing the video to be viewed at any time. In Togo, (who received a copy of the video before the start of their campaigns) the video was used in pre-campaign training. In Ghana, the information from the video was used to inform the training but the video was not shared with drug distributors.

### Evaluation case study in Guinea

The use of the video job-aids was evaluated in two districts, Siguiri (Doko subdistrict) and Labé (Popodara subdistrict), in Guinea during and after the last SMC cycle of the 2020 SMC campaign, between October and December 2020. The aim was to understand how the video had been used, what participants had understood and put into practice from the video, cultural and other barriers, and the potential value of a video to train drug distributors. A rapid ethnographic technique was employed, using in-depth interviews (IDIs) with the national coordinator and with district SMC coordinators and SMC supervisors, focus group discussions (FDGs) with drug distributors, direct observations and informal conversations, led by two Guinean researchers from the Centre National de Formation et de Recherche en Santé Rurale de Maferinyah, Forécariah, Guinea. IDIs focused on how the video was shared, its use by drug distributors, and how the video could be used in future during training sessions at the start of the SMC campaigns. FDGs (three in Doko and three in Popodara) with drug distributors (10–12 distributors per group), considered the use of the video and the extent to which the content of the video was assimilated and put into practice by distributors. Direct observations of SMC distribution, (in Doko only), were undertaken with eight pairs of drug distributors, to understand local distribution practices and the nature of interactions with household members. Along with the observations, researchers had informal conversations with household members, other villagers and distributors about the SMC process and experience during COVID-19.

IDIs and FGDs were held in French, whereas French and the local languages (Maninka, Pular) were used where needed during informal conversations.

FGDs and IDIs were transcribed in French and, with fieldnotes from observations and informal conversations, analysed following a thematic approach with deductive and inductive coding, using Nvivo software, version 11. Deductive codes, derived from the literature and experience of the researchers, were first applied to the data. Then, inductive codes—those emerging from the IDIs and FGDs—were added. Codes were then grouped into categories, in line with the specific research questions, and information from direct observations and informal conversations was triangulated with that from IDIs and FGDs. The data were interpreted taking into account the cultural context and COVID-19 situation in the study districts.

### Workshop with national malaria programme managers

Two workshops were held in January 2021, one in English and one in French, with the national coordinators and other stakeholders who organise or deliver training to SMC drug distributors. The aim of the workshop was to explore how the video had been used during the 2020 SMC campaign, the extent to which the new training video enabled drugs distributors to operate safely and effectively during the COVID-19 pandemic, and to identify the wider lessons for the training of drug distributors and for the future use of video job-aids for community health workers in sub-Saharan Africa. Each workshop was held online over two hours. Before each workshop, a worksheet was sent in advance to each NMCP team with links to the videos (S3 File). These sheets were meant to be completed during the workshop and sent back organising team afterwards. Further informal conversations added insights to countries’ use and perceptions of the video. The information collected was used to inform updating the videos for the 2021 campaigns.

### Ethics

Documented verbal consent was obtained for the IDIs, FGDs and direct observations, including permissions for the use of anonymous quotes, after explaining the aims and process of the study in the local language. Information sheets in French were given to the participants, explained by the interviewer in the local language (in the presence of a witness for participants who were unable to read), and verbal consent was documented on paper forms signed by the interviewer with the participant’s name, village of residence and date. For the focus groups, participants were contacted before the session to seek consent, which was reconfirmed on the day of the focus group. For the direct observations in households, the SMC drug distributor asked the head of the household and /or caregivers if the researcher could observe the SMC administration. If they agreed, the researcher then explained the study to the household members, including that they may take notes and ask questions as well and obtaining verbal consent from the head of the household. With permission, IDIs and FGDs were audio recorded. All personal identifiers were kept confidential and each interview was given a coded number which corresponded to initials and job title. Participants in the online workshops gave verbal consent for the information shared during the workshop to be used in this evaluation.

Ethical approval was obtained from The London School of Hygiene and Tropical Medicine, UK (ref 22698), and by the National Ethics Committee for Health Research (CNERS) in Guinea (ref. L-148-CNERS-20).

## Results

### Views of malaria programme managers on advantages and limitations of video resources in SMC training

In total, 52 participants from 11 of the 13 SMC countries participated in one of the two workshops held in January 2021, in English for The Gambia, Ghana and Nigeria, and in French for Benin, Burkina Faso, Cameroon, Guinea, Mali, Niger, Senegal, and Togo. Participants were staff responsible for coordination and training of SMC drug distributors. Before the workshop, all participants were sent a link to the latest version of the video, and were asked to view the video before the workshop.

Many advantages were identified for using the video for training: the messages are clear and simple, and easy to remember, and the video conveys the same information to everyone. The video could enable training without the need to gather in-person, with benefits in saved time and cost in addition to maintaining social distancing. The videos could also potentially be used in online training. The videos can be viewed on android devices used in the field, as a job-aid providing a reminder of the delivery process both for distributors and supervisors, and as an aid to supportive supervision.

The main challenges with using the video for training were practical (phones, power, internet, data projectors); there were concerns about language and accent of the audio narration impacting on understanding; and differences between the video and national practices with respect to details of the procedures for administering SMC drugs. If the video is used without in-person training it could be difficult to ensure everyone has participated and is able to ask questions to improve understanding.

The advantages and limitations of videos for training that the workshop identified are listed in Table 2.

**Table 2:** Advantages and limitations of using the videos for training.

Advantages	Limitations
The videos:	Challenges include:
**1**. provide a practical demonstration of the process of administering SMC safely and recording the information	1. ensuring everyone has engaged with the video. When the training is not in the classroom, it can be difficult to ensure that everyone has participated in the training
**2**. provide simple clear messages	2. lack of opportunities to ask questions
**3**. ensure everyone has the same information	3. not all drug distributors have an android phone so access to the video may be difficult
**4**. audio visuals are useful teaching aids as the images stay in people’s minds and help with learning and retention of information	4. not all areas have power or data projectors to show the video to a group during in-person training
**5**. can be used to train drug distributors and as an aide memoire by drug distributors and supervisors	5. narration needs to be in local languages and accents
**6**. can be shared on electronic platforms	6. videos need to reflect local practices and national guidelines, and terminology
**7**. could be used for online training, and reduce the need for travel	
**8**. are short and save time	
**9**. provide a useful summary of the content of in-person training	

### Opportunities for using the video in training

Workshop participants reflected on opportunities for making use of the videos during training. The video could be given to participants in advance of the training, to familiarise them with the information; face-to-face training could utilise role-plays, which could be based on situations presented in the video. Additional short videos could show challenging situations that could be used to reflect with trainees about effective ways to deal with such situations. After face-to-face training, the video is useful to remind distributors of what they need to do, as the SMC campaign can extend over 5 months, and staff or volunteers may also work on other campaigns during this period. Stills or images from the videos could be used in in-person training in areas with poor internet connectivity or lack of electricity, to demonstrate practice or stimulate debate. The videos could be used in a number of different ways: shown to trainees during in-person training or used to identify messages to be incorporated into face-to-face training, and to provide a focus for assessment of understanding.

### Case study in Guinea

Drug distributors who watched the video stated that it reflected what they had been taught in previous training but the information in the video was easier to understand and more practically oriented than the in-person training they had received.

" *It’s a reminder tool*, *and I call it an aide-memoire that the [distributors] can use so as not to make mistakes"*

IDI, Regional Health Officer

Supervisors felt the video could support their work, drug distributors could refer to the video in the first instance to check the procedures, without needing to ask the supervisor.

"*… it will also facilitate the work of the supervisors*. *A supervisor who has to supervise several shifts*, *and if this video is available [with distributors]*, *this may decrease supervisors’ workload* "

IDI, SMC national supervisor

Some distributors in Labé said they generally followed all the steps mentioned in the video, but many other distributors in Labé and in Siguiri acknowledged that, although they knew the correct procedures, they did not observe all the video’s recommendations. Distributors said that respecting all the steps recommended in the video was time consuming, therefore following all the steps was not practical in terms of the target number of children to cover with SMC per day. Each pair of distributors are expected to cover 80 children during an eight-hour day, which is an average of six minutes per child. The shortest time observed for a single administration was four minutes, and the longest time sixteen minutes, and these figures do not include time to move from one household to the next. The shorter administrations, were instances where the child’s caregiver accepted the SMC immediately without extended discussion, and the child was present. In longer administrations, which were the majority, either the caregivers had a long discussion with the distributors before agreeing, or the child was absent or could not take the drugs immediately (playing, sleeping or had to eat first). Given the daily targets of 80 children, distributors would often not follow all the procedures as they are time consuming.

*"You know that we [are taught] everything [all the recommendations] during the training*, *but in the field it’s different*, *we can’t respect everything*. *If you respect all the instructions*, *the procedures as they say in training*, *you’re going to delay a lot*, *and if you come back from the field in the evening*, *they’re going to scold you that you’re not working well*. *It’s very tricky indeed…"*

Informal conversation, SMC distributer, male, Labé, 13th December 2020

The procedures that should be followed to deliver SMC drugs safely during the COVID-19 pandemic, were not always followed. Adherence to these procedures was problematised in the Guinea context due to mistrust of health workers and in disbelief in the existence of the COVID-19 virus. There was widespread mistrust in Guinea of government healthcare services and workers following the 2013–2015 Ebola crisis [10,11]. This mistrust has been reactivated with the COVID-19 pandemic, and many community members do not believe in the existence of COVID-19, or its presence in their locale. Drug distributors were observed not using masks in Siguiri and a very few wearing them in Labé. Indeed, community members, especially in Siguiri, perceived the use of face mask as the signal that the COVID-19 is present, and some community members suspected distributers to spread the corona virus. It was therefore felt that requiring drugs distributors to wear face masks would be received badly and result in lower rates of take up of the SMC drugs. Some parents said they refused SMC because they did not recognise the drug distributors with a mask.

"*The [distributors] who came here the first time were all wearing masks*. *When they came*, *I couldn’t recognise them with the mask*. *I did not want to accept what them [their service] because I could not know who they are*. *But when the girl [one of the distributors] took off her mask*, *I immediately recognised her and finally*, *I allowed them to give the drugs*"

Informal conversation with a Caregiver

*"…I have my face mask in my packbag*, *but as you know it is not cautious to take it out…"*

Informal conversation, SMC distributor

Some drug distributors found it hard to follow the guidance on social-distancing in the video, physical distancing when visiting a household, in particular towards those who are older than the distributor, can be considered disrespectful.

*“…people sometimes*, *if you distance yourself from them*, *they will feel offended*, *and it is difficult to observe social distancing; shaking hands is a much-appreciated custom and to fail to do so is to disrespect the elders”*

Informal conversation, SMC distributor

A key aspect of the social distancing guidance was that the distributor should pass the blister pack to the caregiver, and ask them to administer the medication while observed by the distributor, rather than the usual practice which was that the distributor administers the medicine. In Guinea, distributors were advised not to let the caregivers suspect that this change was due to COVID-19. Instead they were told to explain this change so that caregivers could learn to administer the SMC whilst being observed, so that they know how to do this when they administer the remaining doses unsupervised on each of the next two days.

“*We said again*, *we must not tell the parents that we ourselves do not give the medication because there is Covid-19*, *that we must not say that*. *That we must say*, *as we do every year*, *so you are going to distribute this time*, *we will see if you have understood how we do it*.*”*

SMC Drug distributor

Distributors were issued with alcohol-based hand rubs, or ‘gel’ rather than needing to use soap and water, but were advised to use the gel out of sight of the community. The video showed distributors asking for soap and water to wash their hands, but in Guinea this could be considered impolite especially if asking an older member of the household. Some distributers therefore preferred not to ask, and so did not wash their hands before providing the SMC drugs. The video was adapted to remind the drug distributors to wash their hands at the start but the instruction to ask households to provide soap and water was omitted. This allowed the drug distributors to choose how best to clean their hands in a given context.

## Discussion

Smart phones are increasingly used in community health programmes [5,12–17], but the use of video job-aids that can be displayed on smart phones or android devices has not been widely exploited. We investigated the use of video job-aids to support the delivery of seasonal malaria chemoprevention (SMC), and showed that the approach has many advantages. The videos convey the same information to everyone, and the audio-visual images stay in people’s minds and help with learning and retention of information.

The videos were short and could be distributed easily via Whatsapp or Bluetooth, with the potential to be cascaded to large numbers of distributors in a short time, and not requiring significant battery power to be expended during viewing, so that they could be used in the field during delivery as a job-aid, as well as during pre-campaign training.

Careful consideration was needed in defining the video content, to ensure it reflected WHO guidelines and consensus good practice, while also recognising certain differences in procedures country to country. Piloting content is an important step to ensure content is consistent with cultural norms and is not overly prescriptive with respect to actions that may need to be adjusted in particular settings. For example, in Guinea, some COVID-19 safety measures could not always be followed due to negative perceptions by communities.

Some community members thought that SMC distributors may bring COVID-19, or wished to test for COVID-19. In Nigeria, the SMC teams were also charged with looking for COVID-19 and reporting any suspected cases and this created some mistrust. However, methods that improved trust and helped distributors to adhere to safety measures in the video included, in The Gambia, training on how to communicate to the community about the importance of wearing a mask, and in Ghana, the use of volunteers well known in the local community to delivering SMC.

Most drug distributors are members of rural communities and may not speak the official national language. Video versions were needed with narration in a range of languages to improve understanding. The accent may also be important. In Togo, the French accent in the video was difficult to be caught by local people, and so ideally the video needs a Togolese French accent. It has been estimated that out of 1200 different languages spoken in West Africa, 130 are commonly used among 80% of the population [18]. Voice-overs in different languages and accents could be added in each country, this would increase engagement with the video and promote ownership of the messages, and strengthen local capacity to produce tailored video resources.

Limitations of the video job-aids include that when the training is not in the classroom, it can be difficult to ensure that everyone has participated in the training, and there is no opportunity to ask questions. However, local coordinators can use the videos to enhance supportive supervision and can respond to questions. Videos can be effective in complementing classroom training [19], the SMC video can be shown during in-person pre-campaign training, and form the basis for group discussion.

A limitation of our evaluation was that not all distributors had a smart phone. However, SMC programmes are increasingly using ‘digital’ delivery whereby distributors are provided with android devices to track delivery, these devices can be used to view the videos.

During the 2020 campaigns, three countries used digital data collection, The Gambia, Ghana and Benin. In 2021, a further three countries piloted digitalisation, Nigeria, Burkina Faso and Guinea [2], with other countries expressing interest of using these tools for future campaigns. Therefore, the lack of smartphones/tablets should not be seen as a long-term barrier to uptake, as video resources can increasingly be viewed on tools provided by the SMC campaign. We were not able to assess the likely uptake of the video among distributors and did not assess systematically the learning from the videos, or the impact of the video on quality of delivery. Such evaluations could be undertaken in areas where distribution is undertaken using android devices.

We used animation rather than live action, this has the advantage of being effective across diverse cultural groups with only the voice-over needing to be modified for different audiences. The animation depicted key messages clearly through the images as well as through the narration. There is evidence that watching aminated videos can enable better retention of information than face-to-face training [17].

SMC involves door-to-door distribution during 4 to 5 months each year to over 30 million children in 13 countries, by tens of thousands of drug distributors. Ensuring quality of drug administration on such a scale is a major challenge. We have shown that video job-aids can be used to promote adherence to good practice guidelines but care is needed to ensure content is appropriate to the local context. Their use in community health programmes should be more widely evaluated.

## Supporting information

S1 File Video production. (DOCX)

S2 File Video scripts in English, French, Hausa, Fula and Portuguese. (DOCX)

S3 File Workshop worksheet. (DOCX)
