# Supplementary material for: The use of video job-aids to improve the quality of seasonal malaria chemoprevention delivery
Source: PLOS Digit Health. 2022 Dec 22;1(12):e0000165. doi: 10.1371/journal.pdig.0000165 (PMC9931299; doi:10.1371/journal.pdig.0000165)
Supplement: S1 File — (DOCX) [file pdig.0000165.s001.docx]

**The use of video job-aids to improve the quality of Seasonal Malaria Chemoprevention delivery**

Susana Scott, Bienvenu Salim Camara, Michael Hill, Eugène Kaman Lama, Lansana Barry, Aurore Ogouyemi-Hounto, William Houndjo, Gauthier Tougri, Nombre Yacouba, Dorothy Achu, Marcellin Ateba, Mahamat Saleh Issakha Diar, Keziah L. Malm, Kofi Adomako, Paolo Djata, Wica Da Silva, Idrissa Cissé, Vincent Sanogo, Hadiza Jackou, Nnenna Ogbulafor, Bala M Adu, Jamilu Nikau, Seynabou Gaye, Alioune Badara Gueye, Balla Kandeh, Olimatou Kolley, Tinah Atcha-Oubou, Tchassama Tchadjobo, Kovana Marcel Loua, Andre-Marie Tchouatieu, Ibrahima Mbaye, Maria-Angeles Lima-Parra, Abena Poku-Awuku, Jean Louis Ndiaye, Corinne Merle, Liz Thomas, Paul Milligan

**S1 File: Video production**

Table of Contents

[Table A: Stages of video production 2](#_Toc117766069)

[Table B: Country specific adaptions for the SMC video job-aid 3](#_Toc117766070)

## Table A: Stages of video production

| **Pre-production** | **Production** | **Post -production** |
| --- | --- | --- |
| - Aims, purpose and target audience defined - Scope and content defined - Video style agreed - Draft script in English and French, reviewed by advisory group and revised | - Storyboard created, reviewed by an advisory group and revised - Animations developed - Editing to keep length of film under 6 minutes - Animation version with French text - Sent to NMCPs for review - 2 content versions of the video developed in line with feedback from NMCPs* - Script translated into Hausa, Fula, and Portuguese, translations validated, and narrations recorded | - Addition of video introduction by NMCP in some countries - The video uploaded to Vimeo, YouTube channels and project websites with a range of download options ranging from high resolution (1920x10180, 74.9Mb) to low resolution (640x320, 21.4Mb) - Final version sent to NMCP via WhatsApp to be cascaded down to SMC teams - Evaluation by NMCPs - Further content versions developed |

**NMCP: National Malaria Control Programme*

## Table B: Country specific adaptions for the SMC video job-aid

| **Variations from country feedback** | **Country** | **Adapted for the 2020 campaign** | **Adapted for the 2021 campaign** |
| --- | --- | --- | --- |
| Wearing mask throughout the video (instead of previously putting them on in front of the household | All countries | yes | yes |
| Administering the SMC drugs via spoons rather than cups | Guinea Bissau  Benin | yes | yes |
| SMC Age range extended to 10 years of age | Senegal | yes | yes |
| More local languages | All countries | Hausa narration | Fula narration |
| More local accents | Togo | no | yes |
| Showing digital data collection as well as paper collection | Ghana  The Gambia  Benin  Nigeria (2021 campaign)  Burkina Faso (2021 campaign)  Guinea (2021 campaign) | no | Yes |
| House marking when household has had SMC delivered | Ghana | no | yes |
| Marking child’s fingers once they received SMC | Ghana  Cameroon  Burkina Faso  Mali | no, due to COVID-19 restrictions | no, due to COVID-19 restrictions |
| Wristbands on children once they received SMC | Guinea Bissau | no, due to COVID-19 restrictions | no, due to COVID-19 restrictions |
| 3-day supervision versus 1^st^ day only | Burkina Faso  Benin  Senegal | no, due to COVID-19 restrictions | no, due to COVID-19 restrictions |
| Community health worker tests for malaria and treats if positive during the SMC campaigns | Mali  Togo | no | no |
| Malnutrition screening done at during the SMC campaigns | Mali  Niger  Burkina Faso | no | no |
