# Supplementary material for: The use of video job-aids to improve the quality of seasonal malaria chemoprevention delivery
Source: PLOS Digit Health. 2022 Dec 22;1(12):e0000165. doi: 10.1371/journal.pdig.0000165 (PMC9931299; doi:10.1371/journal.pdig.0000165)
Supplement: S2 File — (DOCX) [file pdig.0000165.s002.docx]

**The use of video job-aids to improve the quality of Seasonal Malaria Chemoprevention delivery**

Susana Scott, Bienvenu Salim Camara, Michael Hill, Eugène Kaman Lama, Lansana Barry, Aurore Ogouyemi-Hounto, William Houndjo, Gauthier Tougri, Nombre Yacouba, Dorothy Achu, Marcellin Ateba, Mahamat Saleh Issakha Diar, Keziah L. Malm, Kofi Adomako, Paolo Djata, Wica Da Silva, Idrissa Cissé, Vincent Sanogo, Hadiza Jackou, Nnenna Ogbulafor, Bala M Adu, Jamilu Nikau, Seynabou Gaye, Alioune Badara Gueye, Balla Kandeh, Olimatou Kolley, Tinah Atcha-Oubou, Tchassama Tchadjobo, Kovana Marcel Loua, Andre-Marie Tchouatieu, Ibrahima Mbaye, Maria-Angeles Lima-Parra, Abena Poku-Awuku, Jean Louis Ndiaye, Corinne Merle, Liz Thomas, Paul Milligan

**S2 File. Transcripts of the narration for the SMC video job-aids**

Table of Contents

[Northern and Southern FULA Transcripts 2](#_Toc117765825)

[Hausa transcript: Matakan Bada Maganin Zazabin Cizon Sauro A Lokacin Damuna  Wato SMC 14](#_Toc117765826)

[French transcript : Administration de la Chimioprévention du Paludisme Saisonnier en porte à porte (5 ans) 19](#_Toc117765827)

[Portuguese transcript: Administração porta a porta da Quimioprevenção do Paludismo Sazonal (QPS) 23](#_Toc117765828)

[English transcript: Delivering Seasonal Malaria Chemoprevention (SMC) 27](#_Toc117765829)

**
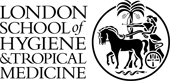

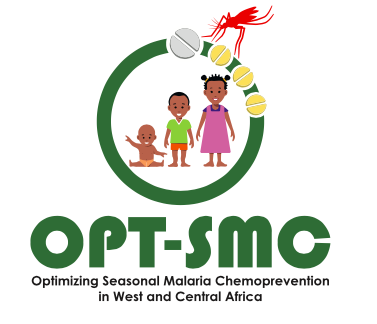
**

**OPT-SMC Training videos**

Delivering Seasonal Malaria Chemoprevention (SMC)

## Northern and Southern FULA Transcripts

*To note: Narration assumes COVID restrictions will be in place, distributors recommended to wear masks, maintain social distancing, and to pass the medicine to the caregivers to administer under observation.*

*Video with COVID messages and both paper and electronic recording, using cups or spoons (*[*https://www.lshtm.ac.uk/research/centres-projects-groups/opt-smc#smc-training-videos*](https://www.lshtm.ac.uk/research/centres-projects-groups/opt-smc#smc-training-videos) *)*

|  | **Transcript for Cups** | **French** | **Fula (1) - includes Guinea, Cameroon and parts of Senegal and Gambia** | **North Fula (2) – includes large parts of Senegal and Mali and parts of Nigeria** |
| --- | --- | --- | --- | --- |
| 1 | After watching this video you will understand how to deliver SMC, to help protect children against malaria during the rainy season. | Après avoir visionné cette vidéo, vous apprendrez mieux comment se déroule la campagne annuelle de CPS et ce que vous devriez faire pour protéger les enfants contre le paludisme pendant la saison des pluies | Toumah gai nou n’ong ndarou deh ooh video, ung faa’ma koh hon’no yot ti’nirr tong kah CPS, koh wal’lah dan dou deh soukabeh nyaw malaria (mbou’ti) kah nder ndoungou. | Sika gayni ndarou deh ooh video, ma on beydo faamoudeh hono campagne CPS oh yaarat eh hono mbadata ha dandaa soukaabeh hé jontinoodji si ndoungou ari |
| 2 | This year, because of COVID19, all health workers should wear a mask, and avoid close contact with anyone in the household. | Cette année, du fait de la COVID19, tous les agents de santé doivent porter un masque et garder une distanciation physique avec tous les membres du ménage~~.~~ | Hik’kah fii nyaw Covid, kah lah gol’loh woh noh douah wat’tah deh mask (sou’ntah houndou koh knong), mb’eh pot’tor ndira kah soo’dou | Hik’kah, sab Coronavirus, kala gollotodo he thiellal ena foti waddeh mask beh ngod’dodira hé yimbeh gallé beh |
| 3 | When you arrive at a household, explain you and your co-worker have been sent by the district health team to deliver important medication that helps to prevent children from getting sick from malaria. | Dès que vous arrivez dans la concession, expliquez que vous et votre collègue êtes mandatés par le district, vous êtes venus pour dispenser un traitement important qui protégera les enfants contre le paludisme. | Si’ong hewtii kah soo’dou, aanding yim’beh beng ang ehmoh golida’tah onong koh santee ong naylii ong yoh ong yoot’ting lek’key koh dandah sou kah beh beng wot’tah beh heb’bew nyaw mbou’ti (malaria). | Si on ndiottima hé gallé, fammine beh kou gallé doctor oh neli on yone yottine lekki ngam dandoudé soukaabeh hé jontinoodji |
| ~~4~~ | Make sure you keep a safe distance from the care givers and children at all times. | Assurez-vous de garder une distanciation physique par rapport à la gardienne de l’enfant et aux enfants | Et’todeh pot’tor ndirong eh gol’loh beh beng kah nyoung eh sou kah beh beng | Et’todeh ngoddondirone hé kalfanaado sukaabeh hé hakkoundémon hé soukaabé |
| 5 | Wash your hands | Laviez les mains | Loteh judeh mong ndeng | Looteh juudé mon déh |
| 6 | Now you’re ready to start.  Find a place to sit and ask for a cup, a spoon, and clean drinking water | Vous pouvez à présent commencer  Trouvez une place pour asseoir et demandez à la gardienne d’enfant d’apporter une tasse, une cuillère, de l’eau potable | Jooni koh fou’nta gol hed’di  Ndambeh kah joh doh ntah, eh kass, coudou eh diyang laa’bou n’tang koh yaree | Jooni ada waawi fudaadeh golleh  Ndaar tu ndiodiida,  Naamno kaas hé koudd hé ndiyam |
| 7 | Set out the things you will need:  ✓ The SMC blister packs  ✓ SMC record cards  ✓ If you are recording SMC treatment digitally, you will need to open the App on your android phone or tablet  If you are not using digital recording you will need:  ✓ the SMC Register  ✓ and the Tally sheet | Sortez vos outils pour la procédure.  Cela comprend:   - La plaquette de comprimés CPS - La carte CPS   Si vous enregistrez le traitement de la CPS électroniquement, vous devrez ouvrir l'application sur votre téléphone ou tablette Android  Si vous n’utilisez pas de l’enregistrement électronique, vous aurez besoin :   - Le registre ou carnet - La feuille de pointage | Wal lineh koh faalah n’tah kong   - Packetaagy n’teng - Kaideejeh SMC   Sii aa windaii kah nder machine, aa ouditaii App ong kah nder telefong mah mah koh winderr the kong  Sii wonah nong,   - Kaideeri SMC ndeng - Kaideeri jogyndeh lek’koikoi | Yaltino gollordé ma  Udum tongui ku :   - Pakedaaji podd’é - Cartal CPS - Sika windiino hé nder machine ma, a oudditata app o hé nder telephon o.   Si wana doum non   - Deftere walla Karni CPS - Kaayit joguib’é lekki |
| 8 | If this is the first visit of the year, you will need to record details of ALL children under 5 years of age, in the SMC register, or on your device. So ask to see these children.  If a child is under three months, he or she will NOT receive SMC medication during this visit, but they may later in the year, so make sure you register them.  This helps to ensure children are not missed when you return each month. | S’il s’agit du premier passage de l’année, vous devriez recueillir, dans le registre CPS sur votre tablette ou téléphone android, les informations de TOUS les enfants âgés de moins de 5 ans, alors demandez à voir tous ces enfants.  Si un enfant a moins de 3 mois, il ne recevra PAS le médicament de la CPS lors de cette visite, mais il pourra le recevoir plus tard dans l’année. Assurez-vous de l’enregistrer dans le registre.  Cela permet de s’assurer que les enfants ne manqueront pas le traitement lors de vos prochaines visites. | Sii koh lawol aaranong ngol nder heetandeh ndeng, aa windaii fii souk’kah beh beng fow beh hebaani beng doubi johwee nder kaidee mah kah macin. Landoh yee ah souk’kah beh beng.  Sii souk’kah ong hebaanii leb’bi ta’ty, ooh hebaa’tah kii lek’kii ngol doh lawol, koh noh nowawii yea soh kah nder hiita’ndeh. Awah windu beh kah kaideerri  N’ton wal’laii hara souk’kah beh beng fow nyaw ndotoh sii aa yiltikeh lewru woh lewru. | Siko laawol adiingol hé hitaandé hé, A windat kabaaruujé sukaabé bé suwa b’ennudé duubé 5 foh hé karni CPS walla hé telphon hé walla hé machin hé  Si tawi soukaabé kébaani taw lebbi  Tati (3), o hébata lekki ngoldo laawol, kon non ena waai yeso hé nder hitaandé hé o héba . Ada waawi windoudémbo hé  Ena walla ha soukaabé beh mbaasa yejjiteedeh niandé wond’dé |
| 9 | You will also complete an SMC record card for each eligible child. | Vous devez remplir également une carte CPS pour chaque enfant éligible. | Koh Gainougol windougol kaidee souk’kah ong | Ad’a foti rampildé kartal CPS bu kal suk’kah |
| 10 | Write the child’s name and personal details. You will record the treatment AFTER you have given the SMC medicine. | Reportez le nom, prénom et les données personnelles de l’enfant. Vous ne reportez le traitement CPS qu’APRES avoir administré le médicament CPS à l’enfant | Windou indeh souk’kah ong, honto ooh heb’bah, mawbeh mak’koh beng. Ong windaii lek’kii koh jon’nou n’tong moh kong | Windu inndé hé yettoodé suk’kah o  A winndata lekki ki suwana suk’kah o yari lekki ki haa gasi |
| 11 | Before you begin, you need to determine if the child is eligible for SMC. | Avant de commencer vous devriez déterminer si l’enfant est éligible à recevoir le traitement de la CPS | N’toh eh ong fou n’tadeh, hee n’tong douaa n’darru deh sii souk’kah ong noo douaa hebbu deh kii lek’kii | Had’ma fud’daadé, ada foti yeewdé ngam suk’kah o ombo foti heb’dé lekki CPS ki |
| 12 | First, establish how old the child is.  If the caregiver is unsure of the child’s age, ask to see the vaccination card, or ask other members of the household. | Premièrement, vous devez déterminer l’âge de l’enfant  Si la gardienne de l’enfant doute, demandez à voir le carnet de vaccination de l’enfant ou demandez à un autre membre de la famille de vous renseigner | Aaranou – koh dou bii jeelou souk’kah ong heb’bi  Sii joogee domoh ong andah, landoo mewbeh mak’koh ben mah n’dara kaidee jibinandeh mak’koh n’deng | Ku adi fof, ada foti ndaarudé duubé suk’kah o  Sika laabaani mawdo mak o, ndaru karni niakku suk’kah o, mbar namno da gooto hé yimbé gallé o |
| 13 | If the child is unwell they cannot take SMC now. They must go to the health centre or to a community health worker so the child can be tested. | Si l’enfant ne se sent pas bien, il ne peut pas recevoir le médicament CPS, il doit être conduit au centre de santé ou à un agent de santé local afin d’être examiné. | Sii souk’kah ong sell’ah, wot’tah ooh jeetou lek’kii king jooni. Yoh oh yah kah lob’itan (san’tee) mbeh n’dara moh | Si suk’ah o sellani, o fotani yardé lekki CPS ki. Yoh oh yah to hopital hé (san’tee) mbeh n’dara moh |
| 14 | If they have malaria, they will be given A-C-T treatment. | Si l’enfant a un test positif pour le paludisme, il doit recevoir une C-T-A. | Sii mbeh heb’bi nyaw mbou’ti, mbeh nyaw ndeee teh A-C-T | Sika o jeewaama ha tawi o hebi jontinoojé, Omba foti yardé lekki A-C-T |
| 15 | If the child has had severe side effects from SMC, or from other medicines, in the past, they must not take SMC. | Si dans le passé l’enfant a eu des effets secondaires sévères dus aux médicaments CPS ou à d’autres médicaments, il ne doit pas recevoir de médicaments CPS. | Sii souk’kah ong nawnaii sii oh getti lek’ki king ma’ah lek’keh leh awah wot’tah ooh jeet’tou lek’kii SMC | Sika sukah o meed’i heb’dé tiadeelé tiattudé tiaggal ndo yarinoo lekki CPS ki walla lekki ngokki, o fotaani yardé laawol gongol CPS |
| 16 | Ask if the child has taken other medicines in the last four weeks. | Demandez si l’enfant a pris d’autres médicaments au cours des 4 dernières semaines. | Lan doh sii souk’kah ong jet’ti lek’kii eh n’derr jonteh naii m’bawoh | Naamno mbar suk’kah o meda yardé lekki hé ndeer jonti neyo (4) bennudé |
| 17 | If they have taken SP, or AQ, (for malaria) or Bactrim or Cotrimoxazole (for an infection), SMC must not be given today. | Si l’enfant a pris de la SP ou de l’AQ (pour le paludisme) ou du Bactrim /Cotrimoxazole (pour une infection), il ne devrait pas recevoir le médicament CPS de ce jour | Sii beh jet’ti n’deh lek’kelleh SP or AQ fii nyaw mbou’ti maah Bactrim or Cotrimoxazole fii nyaw god’doh, awah wot’tah oh jet’tou lek’kii king handeh | Si suk’kah o hebi lekki SP walla AQ wallaBactrim/Cotrimoxazole, o fotahni heb’dé CPS hé nialawmu hé |
| 18 | Once you are confident the child is eligible, you can proceed. | Quand vous serez sûr de l’éligibilité de l’enfant, vous pourrez poursuivre la procédure d’administration | Sii n’tah hooli souk’kah ong noh douaa heb’bou deh kii lek’kii, awah konting | Si tawi suk’kah o na foti jettudi lekki CPS, ada waawi fud’daadé |
| 19 | Select the appropriate blister pack for the child’s age. | Sélectionnez la plaquette appropriée par rapport à l’âge de l’enfant | Toub’beh doubii souk’kah ong kah kaidee | Sub’ba lekki CPS haandudo hé duub’é suk’kah o |
| 20 | Place the blister pack on the chair or table | posez la plaquette sur un support | Wal’lii neh kaideegi ding kah dow joo’ndorr deh ma’ah kah taabal | Fow lekki ki dow jood’ordé mum |
| 21 | Explain to the caregiver that she will give the first TWO tablets in the pack to the child. | Expliquez à la gardienne d’enfant de l’enfant, qu’elle va donner les DEUX premiers comprimés à l’enfant | Yea toh keeliifah joh yourjonou souk’kah ong kaateh ndedeh lek’kii | Fammina mawdo suk’kah o, yo tottu suk’ah o lek’ki cps didi (2) |
| 22 | Instruct the caregiver to slowly add a small amount of water to fully cover both tablets at the same time. They may have to softly stir the mixture. | Demandez à la gardienne d’enfant d’ajouter lentement une petite quantité d’eau couvrant les comprimés, et de remuer le mélange | Yea’toh keeliifah joh ong yoh waandou kaateh ndeng kah nderr ndeeyang jil’lii ndera hah muncho fii souk’kah ong wahwah yarudeh | Yo kilifa sukah o wada ndiyam seed’a he lekki ki o yongka had’a mako yarnudeh sukah o |
| 23 | Ensure the child is fully awake and sat upright, avoid tipping the head back too far.    Tell the caregiver to administer slowly, checking the medicine has been swallowed.  Tell them to rinse the cup with a small amount of water and give to the child to swallow again, making sure the cup is now empty.  Wait for a few minutes to ensure all is well with the child. | Assurez-vous que l’enfant est bien éveillé et assis en position droite. Veillez à ce que la tête ne soit pas trop penchée vers l’arrière.  Demandez à la gardienne de donner lentement le traitement à l’enfant en s’assurant que tout le contenu du verre a été avalé.  Demandez à la gardienne de verser un peu d’eau dans le verre et de donner le contenu à l’enfant en s’assurant que le verre est maintenant vide  Patientez quelques minutes pour s’assurer que tout va bien pour l’enfant | E’toh deh haarah souk’kah ong ndanaaki, heemoh joodi harah ong yil’tii nani hooreh mak’koh ndeng mba’woh hah  Yea toh keeliifah joh ong yoh jon’nou moh saydah saydah, nohndar tii ndoh souk’kah ong moh’tee lek’kii king. Yea toh beh yoh beh law ju goubouleetii ong, joona souk’kah ong ndeeyang yarah ooh moh’tah kaadii, lan’nah ndeyang n’tang.  Hab’boh tah saydah ndartii noh tah souk’kah ong noh eh djam | Ethodheh yewdo mba suk’kah o dhaanaaki taw omba joodi jondeh mojereh    Yo taw kilif sukah o ombo totumba lekki ki seedah seedah ha ki gassa  Si gassi lallitah coppu o haa laaba  Caggal ndu suk'kah o yari lekki ki ha gassi, sabbo foddé minitaadji seeda ha laabéd'a suk'kah o diambotaani haydara. |
| 24 | Tell the caregiver if the child vomits ALL the medicine in the next 30 minutes, they can be redosed. They will need to find you and ask you to come back, so you can give the caregiver a new blister pack. Remember to retrieve the old one, to dispose of later.  If the child vomits again, note the event in the register or in the App on your device. Do not redose. | Dites à la gardienne d’enfant, si l’enfant vomit TOUS le traitement dans les 30 minutes qui suivent, une deuxième dose de traitement doit lui être remise.  Elle doit vous chercher et vous demander de revenir afin que vous lui remettiez une nouvelle plaquette.  N’oubliez pas de récupérer l’ancienne plaquette pour la remettre à votre superviseur.  Si l’enfant vomit à nouveau, notez l’événement dans le registre ou sur la tablette ou téléphone android . Ne pas lui remettre un nouveau traitement | Yea toh keeliifah joh ong sii souk’kah ong toutii nderr minutagee chaa’pantaaty, yoh jon’nou moh kaadii. Yoh beh arrbeh dam’bii the fii yah jonoubeh lek’kii kaadii nderr packeti keesoh. Ngeti’tou koh jon’nou n’tah beh knog araanou yaa’haa bougoo noh douaa bougorr deh nong.  Sii souk’kah ong toutii kaadii, wot’tah jon’nii tou moh. Yaa windu | Wi kilifa suka’ah o si sukah o tuuti hé nder minutaadji chapantati (30), ya totumbo lekki ngokki.  Yo kilif sukah o noddu neddo santé o ngam lomtindeh lekki ngokki ki.  Sika sukah o tuuti kadi, winda duma hé karni CPS o walla hé macin o walla telephong o. |
| 25 | When you have completed the SMC record card, place it down and step back, so the caregiver can take it. The SMC card is a reminder for the caregiver, and a record of their child’s treatments.  If you are using a tally sheet, mark to indicate how many children have received SMC during this visit. | Après avoir complété le remplissage de la carte CPS, placez-la là où la gardienne d’enfant peut la prendre, et reculez. La carte CPS est un aide mémoire pour la gardienne de l’enfant mais aussi sert à enregistrement le traitement reçu par l’enfant .  Si vous utilisez une feuille de pointage, complétez-la en indiquant le nombre d’enfants ayant reçu le traitement au cours de la visite. | Sii aah gainee windoudeh kaidee ri’nding, wal’ling yil toh ndah mbawoh fii keelifah joh ong jeet’tah kaidee ri’nding. Kaidee ri’nding koh yoh an’ding ting keelifah joh ong honoh souk’kah mak’oh ong wonah eh nywa ndiredeh.  Sii koh lekoi won’tah a knotirrdeh, koh yah windou koh souk’kah beh jeelou yee ou ntah. | Sika rampili cartal CPS ngal, faw duma tu kilifa sukah o wawat dum jettudeh.  Ena wallitah kilifa sukah o hé suftordé, kadi hé windagol likki mbu sukah o jetti.  Sada huutora kaayit tobbateedo, tobbadum ha winda nufoti sukaabé kebi lekki |
| 26 | Explain this treatment is over THREE days. ONE tablet should be taken tomorrow, and the final tablet to be taken the following day. | Expliquez que le traitement complet doit être pris pendant TROIS jours et que l’enfant doit prendre UN comprimé le lendemain et UN comprimé le surlendemain | Yea toh beh nyaw ndou gongol koh nderr bal’deh taatii. Kaateh reh ngete’the jangoo eh faa’the jangoo. | Famminambo suka o nu foti jettudé lekki hé bald’é tati  Handé goto  Jango goto  Mbaawjango goto |
| 27 | Thank the family for taking part in the campaign and remind them of these important messages:  ✓ Remember the date of the next visit  ✓ Keep the pack in a dry place, out of reach of children.  ✓ Finish the blister pack  𝚾 Do not give this medicine to anyone else  ✓ SMC drugs are well tolerated and severe side effects are very rare, but if the child is sick after taking the SMC medicines, take the child to the health facility.  ✓ SMC protects for 1 month, it must be repeated each month, usually for 4 months  ✓ Everyone in your household should sleep under a Long-Lasting Insecticide-treated bed net  ✓ Tell the caregiver to wash their hands once you have left the household. | Remerciez la famille d’avoir pris part à la présente campagne et leur rappelez les messages clés   - Rappeler la date de la prochaine visite - Gardez le traitement dans un endroit sec et hors de portée des enfants - Terminer la plaquette   **X** Ne donnez ce médicament à personne d’autre   - Les médicaments de la CPS sont sûrs et sont bien tolérés. Les effets secondaires graves sont rares. Mais si l’enfant tombe malade après avoir pris les médicaments CPS, amenez-le au centre de santé. - Le traitement CPS protège pour 1 mois. La CPS est essentielle pour une période de 3, 4 ou 5 mois selon la durée de la saison des pluies - Chaque membre du ménage doit dormir sous une moustiquaire imprégnée à longue durée d’action. - Demandez à la gardienne de l’enfant de se laver les mains dès que vous aurez quitté la demeure | Jarrnu yimbeh beng koh beh aarikong aandingtinaa’beh kaadii   - Nyandeh ndeh beh arr tah tah kaadi - Yoh beh maaru kaideegeding kah yoori eh kah souk’kah beh beng hew tah tah - Yohbeh gainou kobeh jonah kong   X wotah beh jog’doh hebu lek’kii king   - Nowahwi sii ongtigy yari lek’ki tampinteh saytah kono hewa, konoh sii souk’kah ong nawnee, nabou moh kah lobtaani (santeh) - Lek’kii king dandaii ontigy lewru wot’turu, ontigy getitaii lek’kii king haa lebbi naii - Mok’kala kah nder soudou sii noh n’tanoo, waaloh toh kah nderr sankeh pompaa’doh - Yea toh keliffajoh ong yoh soh toh joudeh makoh deng sii aa yaltee | Jaarnu yimbé gallé o sabé jeyeedebé hé program o  Suftor naabé   - Niandé wondé nu ngartata - Moftu lekki ki du leppata du sukaabé keb’ata - Yobé ngasnu lekki mbé totta ki - Wotobé totu lekki ki goddo mbu wana jommum - Lekki CPS ki iki siiri iki tampinata. Kono si sukah o niawi chaggal ndu yari dum addembo lopital walla toy santé hé - Lekki ki na danda foddéh lewru. CPS o koka foti nder lebbi 3,4 ha 5, fowi keh lebbi ndungu nu foti hé nukku hé - Yimbé gallo fo nu poti lelaadé hé sankeeji - Yo kilifa sukah o labbina juudé mum sikong gasni |
| 28 | Thank you for watching.    Now you will be able to deliver SMC safely, providing vital malaria protection for children. | Merci d’avoir regardé (suivi) cette vidéo.  Vous êtes désormais capable d’administrer en toute sécurité la CPS pour une protection vitale des enfants contre le paludisme. | Jarama koh ndarru n’tong kong  Jooni ong wawaii joonoudeh kii lek’kii eh jam koh wah watah dandudeh souk’kah beh beng nyaw mbouti. | Jaaraama ku ndaaru don o video.  Jooni ada waawi jonnudé jonnugol siir ngol lekki CPS ki ngam dandudé sukaabé hé jontinoojé |

**
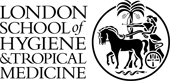

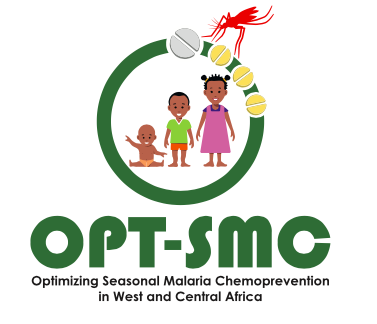
**

**OPT-SMC Training videos 2021**

## Hausa transcript: Matakan Bada Maganin Zazabin Cizon Sauro A Lokacin Damuna  Wato SMC

| S/No. | **Transcript in Hausa** |
| --- | --- |
| 1 | Bayan kammala kallon wannan bidiyo, za a fahimci yadda za a bayar da maganin zazzabin cizon sauro na lokacin damuna mai suna Seasonal Malaria Chemoprevention wato SMC ga yara domin kare su daga wannan cutar. |
| 2 | A wannan shekara, saboda annobar Korona (Covid-19), dole kowanne jami’in kiwon lafiya ya fito a kimtse, ta hanyar sanya safar hanci da kuma gujewa shiga cikin mutanen gidan sosai domin kareyar da wannan cuta. |
| 3 | Da zarar jami’i ya shiga kowanne gida, ya gabatar da kansa da kuma abokin aikinsa a matsayin jami’an lafiya daga hukumar lafiya ta yankin, kuma sun zo domin bada maganin kariya mai muhimmanci daga cutar cizon sauro na yara. |
| 4 | A tabbatar an bada tazara tsakanin jami’ai da kuma masu karbar magani a ko yaushe. |
| 5 | A wanke hannu. |
| 6 | Daga nan sai a fara bada magani.  A nemi wuri a zauna, a kuma bukaci masu gida su bada kofi, cokali da tsaftacaccen ruwan sha. |
| 7 | A fito da kayan aiki kamar haka:  ✓   Katin maganin SMC  ✓   Katin shaidar bada maganin SMC  ✓ **Idan kuna rikodin maganin SMC ta hanyar dijital** bayanan bada magani, dole a bude manhajar a waya ko tabulet.  Iddan ba bukatar dijital a dauki bayanan ba, ana bukatar:  ✓   Takardar rijistar maganin SMC           ✓   da Takardar kirge |
| 8 | Idan wannan shi ne karon ka na farkon zuwa wannan gidan a cikin shekarar nan, ka bukaci ganin dukkan yaran da suke wannan gida yan kasa da shekara biyar kuma a dauki bayanansu a takardar rijistar maganin SMC ko kuma a na’ura.  Idan yaro ko yarinya dan kasa da wata uku ne, BA ZA a ba shi maganin SMC ba a yayin wannan ziyara, amma za a iya bayarwa a ziyara ta gaba. Saboda haka a tabbata an yi musu rijista.  Wannan ya na taimakawa wajen tabbatar da cewa ba a tsallake kowanne yaro ba ko yarinya a ziyara ta gaba. |
| 9 | A tabbata an cika katin bayanan yaran da aka baiwa maganin (SMC Record Card) ga kowanne yaro da ya isa karbar maganin. |
| 10 | A rubuta suna da sauran bayanan yaron. A kuma dauki bayanan bayar da magani BAYAN an gama ba wa yaron maganin SMC. |
| 11 | Kafin a fara bada maganin, a tabbata yaron ya isa a bashi maganin SMC. |
| 12 | Mataki na farko, a tabbatar da shekarun yaron.  Idan iyaye ko masu kula da yaron ba su san ainihin shekarunsa ba, a nemi takardar rigakafin yaron domin tabbatarwa ko kuma a tambayi sauran mutanen da suke cikin gidan. |
| 13 | Kada a baiwa yaron da bashi da ishashshiyar lafiya maganin SMC. A garzaya da shi cibiyar kiwon lafiya ko jami’in kiwon lafiya mafi kusa domin gwaji. |
| 14 | Idan yaron na fama da zazzabin cizon sauro ne, za a bashi kulawar A-C-T. |
| 15 | Idan yaron ya sami wata matsala sakamakon shan maganin SMC ko wasu magungunan daban a baya, to kada a bashi SMC. |
| 16 | A bincika ko yaron ya sha wani magani a cikin mako hudu (4) da suka shude. |
| 17 | Idan yaron ya sha SP ko AQ (na zazzabin cizon sauro) ko kuma Bactrim ko Cotrimoxazole (na kwayoyin cuta), ba za a bashi SMC a wannan rana ba. |
| 18 | Bayan an tabbatar da cewa yaro ya cancanta bayan duba ga matakai na baya, an iya ci gaba da ba shi maganin SMC. |
| 19 | A zabi samfuri ko katin maganin SMC da ya dace da shekarun yaron. |
| 20 | A ɗora katin maganin a kan kujera ko tebur. |
| 21 | A sanar da mai kula da yaron cewa za ta bawa yaron kwayoyi biyun na farkon katin maganin. |
| 22 | Sai a ce ma mai kula su zuba ruwa dan kadan, a hankali kuma yadda zai shanye kan maganin. Za a iya bukatar a dama maganin domin ya narke. |
| 23 | A tabbatar da yaron idon sa biyu, sannan a zaunar da shi da kyau.   A nuna wa mai kula da yaron yadda zata bada maganin a hankali, a kuma tabbatar da yaron ya hadiye maganin gaba daya.  Sannan a dauraye kofin da ruwa kadan, a bawa yaron ya kuma hadiye wa, har sai ya gama shan duka.  Sai a dan jira zuwa wani lokaci domin a tabbatar yaron lafiyarsa kalau. |
| 24 | A gayawa mai kula da yaron cewa idan yaron ya amayar da maganin kafin minti 30 da aka ba shi, za a iya kara ba da maganin.  Su neme ka, ka kuma basu wani sabon katin maganin tare da karbar tsohon domin zubarwa.  Idan yaron ya kara amayarwa a karo na biyu, to a shigar da wannan bayani a manhajar daukar bayanai ta waya ko tabulet. Kuma kar a kara ba shi maganin. |
| 25 | Bayan an kamala cika katin bayanan SMC, a ajiye shi a matsa gefe yadda mai kula da yaron zasu iya daukawa.  Wannan katin SMC na bayanar da mai kula da yaron ne kuma yana dauke da bayanin shan maganin yaron.  Idan kana amfani da takardar kirge, a tabbata an shigar da adadin yaran da aka bawa magani a wannan ziyara. |
| 26 | A yi wa masu kula da yaron bayanin yadda za a bawa yaron ragowar magungunan: Kwaya daya gobe, sannan na karshen kuma jibi. Gaba daya magungun a cikin kwana uku ake shanye su. |
| 27 | A godewa masu gidan domin yarda da suka yi wajen karbar wannan magani kuma a tuna musu wadannan muhimman sakonni:  ✓    Su tuna ranar da jami’an lafiya za su kara dawowa.  ✓   A ajiye katin maganin a wuri mara danshi in da yara baza su iya dauka ba.  ✓  A tabbata an shanye maganin gaba daya kamar yadda aka tsara. Kada a bawa wani daban maganin  ✓ Maganin SMC bashi da illa, amma idan dai har yaro ya nuna alamun samun matsala bayan ya sha maganin, to a hanzarta a kai shi cibiyar kiwon lafiya.  ✓   Maganin SMC yana bada kariya na wata guda ne, wato makonni hudu. Dole a ci gaba da shan wannan magani a watanni na gaba har zuwa wata hudu.  ✓   Kowa a gidan ya dinga bacci cikin gidan sauro mai dauke da magani (mosquito net)  ✓ A gaya wa masu kula su wanke hannayensu da zarar kun fita daga gidan. |
| 28 | Muna muku godiya da kuka kasance tare da mu har zuwa karshen wanna bidiyon.  Yanzu zaku iya bawa yara kyakyawar garkuwa daga ciwon zazzabin cizon sauro ta hanyar maganin SMC. |

**
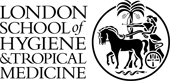

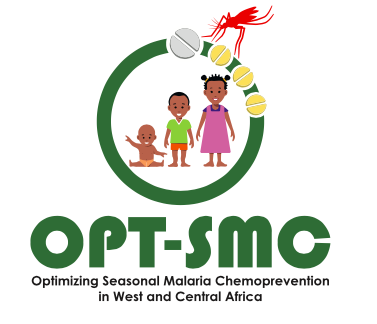
**

**Vidéo de formation du projet OPT-SMC**

## French transcript : Administration de la Chimioprévention du Paludisme Saisonnier en porte à porte (5 ans)

|  | Transcript |
| --- | --- |
| 1 | Après avoir visionné cette vidéo, vous apprendrez mieux comment se déroule la campagne annuelle de CPS et ce que vous devriez faire pour protéger les enfants contre le paludisme pendant la saison des pluies |
| 2 | Cette année, du fait de la COVID19, tous les agents de santé doivent porter un masque et garder une distanciation physique avec tous les membres du ménage~~.~~ |
| 3 | Dès que vous arrivez dans la concession, expliquez que vous et votre collègue êtes mandatés par le district, vous êtes venus pour dispenser un traitement important qui protégera les enfants contre le paludisme. |
| 4 | Assurez-vous de garder une distanciation physique par rapport à la gardienne de l’enfant et aux enfants |
| 5 | laviez les mains |
| 6 | Vous pouvez à présent commencer  Trouvez une place pour asseoir et demandez à la gardienne d’enfant d’apporter une tasse, une cuillère, de l’eau potable |
| 7 | Sortez vos outils pour la procédure.  Cela comprend:   - La plaquette de comprimés CPS - La carte CPS   Si vous enregistrez le traitement de la CPS électroniquement, vous devrez ouvrir l'application sur votre téléphone ou tablette Android  si vous n’utilisez pas de l’enregistrement électronique, vous aurez besoin :   - Le registre ou carnet - La feuille de pointage |
| 8 | S’il s’agit du premier passage de l’année, vous devriez recueillir, dans le registre CPS sur votre tablette ou téléphone android, les informations de TOUS les enfants âgés de moins de 5 ans, alors demandez à voir tous ces enfants.  Si un enfant a moins de 3 mois, il ne recevra PAS le médicament de la CPS lors de cette visite, mais il pourra le recevoir plus tard dans l’année. Assurez-vous de l’enregistrer dans le registre.  Cela permet de s’assurer que les enfants ne manqueront pas le traitement lors de vos prochaines visites. |
| 9 | Vous devez remplir également une carte CPS pour chaque enfant éligible. |
| 10 | Reportez le nom, prénom et les données personnelles de l’enfant. Vous ne reportez le traitement CPS qu’APRES avoir administré le médicament CPS à l’enfant |
| 11 | Avant de commencer vous devriez déterminer si l’enfant est éligible à recevoir le traitement de la CPS |
| 12 | Premièrement, vous devez déterminer l’âge de l’enfant  Si la gardienne de l’enfant doute, demandez à voir le carnet de vaccination de l’enfant ou demandez à un autre membre de la famille de vous renseigner |
| 13 | Si l’enfant ne se sent pas bien, il ne peut pas recevoir le médicament CPS, il doit être conduit au centre de santé ou à un agent de santé local afin d’être examiné. |
| 14 | Si l’enfant a un test positif pour le paludisme, il doit recevoir une C-T-A. |
| 15 | Si dans le passé l’enfant a eu des effets secondaires sévères dus aux médicaments CPS ou à d’autres médicaments, il ne doit pas recevoir de médicaments CPS. |
| 16 | Demandez si l’enfant a pris d’autres médicaments au cours des 4 dernières semaines. |
| 17 | Si l’enfant a pris de la SP ou de l’AQ (pour le paludisme) ou du Bactrim /Cotrimoxazole (pour une infection), il ne devrait pas recevoir le médicament CPS de ce jour |
| 18 | Quand vous serez sûr de l’éligibilité de l’enfant, vous pourrez poursuivre la procédure d’administration |
| 19 | Sélectionnez la plaquette appropriée par rapport à l’âge de l’enfant |
| 20 | posez la plaquette sur un support |
| 21 | Expliquez à la gardienne d’enfant de l’enfant, qu’elle va donner les DEUX premiers comprimés à l’enfant |
| 22 | Demandez à la gardienne d’enfant d’ajouter lentement une petite quantité d’eau couvrant les comprimés, et de remuer le mélange |
| 23 | Assurez-vous que l’enfant est bien éveillé et assis en position droite. Veillez à ce que la tête ne soit pas trop penchée vers l’arrière.  Demandez à la gardienne de donner lentement le traitement à l’enfant en s’assurant que tout le contenu du verre a été avalé.  Demandez à la gardienne de verser un peu d’eau dans le verre et de donner le contenu à l’enfant en s’assurant que le verre est maintenant vide  Patientez quelques minutes pour s’assurer que tout va bien pour l’enfant |
| 24 | Dites à la gardienne d’enfant, si l’enfant vomit TOUS le traitement dans les 30 minutes qui suivent, une deuxième dose de traitement doit lui être remise.  Elle doit vous chercher et vous demander de revenir afin que vous lui remettiez une nouvelle plaquette.  N’oubliez pas de récupérer l’ancienne plaquette pour la remettre à votre superviseur.  Si l’enfant vomit à nouveau, notez l’événement dans le registre ou sur la tablette ou téléphone android . Ne pas lui remettre un nouveau traitement |
| 25 | Après avoir complété le remplissage de la carte CPS, placez-la là où la gardienne d’enfant peut la prendre, et reculez. La carte CPS est un aide mémoire pour la gardienne de l’enfant mais aussi sert à enregistrement le traitement reçu par l’enfant .  Si vous utilisez une feuille de pointage, complétez la en indiquant le nombre d’enfants ayant reçu le traitement au cours de la visite. |
| 26 | Expliquez que le traitement complet doit être pris pendant TROIS jours et que l’enfant doit prendre UN comprimé le lendemain et UN comprimé le surlendemain |
| 27 | Remerciez la famille d’avoir pris part à la présente campagne et leur rappelez les messages clés   - Rappeler la date de la prochaine visite - Gardez le traitement dans un endroit sec et hors de portée des enfants - Terminer la plaquette   **X** Ne donnez ce médicament à personne d’autre   - Les médicaments de la CPS sont sûrs et sont bien tolérés. Les effets secondaires graves sont rares. Mais si l’enfant tombe malade après avoir pris les médicaments CPS, amenez-le au centre de santé. - Le traitement CPS protège pour 1 mois. La CPS est essentielle pour une période de 3, 4 ou 5 mois selon la durée de la saison des pluies - Chaque membre du ménage doit dormir sous une moustiquaire imprégnée à longue durée d’action. - Demandez à la gardienne de l’enfant de se laver les mains dès que vous aurez quitté la demeure |
| 28 | Merci d’avoir regardé (suivi) cette vidéo.  Vous êtes désormais capable d’administrer en toute sécurité la CPS pour une protection vitale des enfants contre le paludisme. |

**
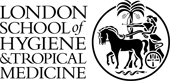

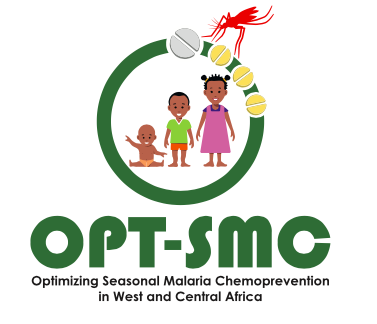
**

**Vídeo de formação do projeto OPT-SMC**

## Portuguese transcript: Administração porta a porta da Quimioprevenção do Paludismo Sazonal (QPS)

|  |  |
| --- | --- |
| 1 | Depois de visualizar este vídeo, saberá melhor como se desenrola a campanha anual de QPS, bem como o que deverá fazer para proteger as crianças contra o paludismo durante a estação das chuvas. |
| 2 | Assim que chegar a uma casa explique que você e o seu colega foram enviados pelo distrito para administrar um tratamento importante que protegerá as crianças contra o paludismo. |
| 3 | Este ano, devido à COVID-19, todos os agentes de saúde devem utilizar máscara e manter o distanciamento físico de todos os membros do agregado familiar. |
| 4 | Garanta que mantém um distanciamento físico em relação à responsável pelas crianças e às próprias crianças. |
| 5 | Lave suas mãos. |
| 6 | Pode começar  Encontre um local para se sentar e solicite à responsável pelas crianças que traga uma colher e água potável. |
| 7 | Prepare o material necessário:  Estes incluem:   - O blíster de comprimidos de QPS - O cartão da QPS - O registo ou boletim - A ficha de contabilização - A caneta |
| 8 | Se se tratar da primeira visita do ano, deve registar as informações de TODAS as crianças com menos de 5 anos no registo de QPS. Por isso, peça para ver essas crianças.  Se a criança tiver menos de 3 meses, NÃO deverá receber o tratamento da QPS nessa visita, mas poderá recebê-lo mais tarde nesse ano. Certifique-se de que a inclui no registo.  Desse modo, garantirá que nenhuma criança ficará sem tratamento nas suas próximas visitas. |
| 9 | Deverá igualmente preencher um cartão da QPS para cada criança elegível. |
| 10 | Escreva o nome da criança e os seus dados pessoais  Apenas deve completar o registo da QPS APÓS ter administrado o medicamento da QPS à criança. |
| 11 | Antes de começar, deve determinar se a criança é elegível para receber o tratamento da QPS. |
| 12 | Em primeiro lugar, deve determinar a idade da criança.  Se o responsável pela criança não a souber, peça para ver o cartão de vacinas da criança ou pergunte a outro membro da família. |
| 13 | Se a criança não se sentir bem, não poderá receber o medicamento da QPS e deverá ser levada ao centro de saúde ou a um agente de saúde comunitária para ser observada. |
| 14 | Se a criança tiver um teste positivo para o paludismo, deve receber o tratamento de ACT. |
| 15 | Se a criança tiver apresentado efeitos secundários graves devido aos medicamentos de QPS ou a outros medicamentos, no passado, não deve receber medicamentos de QPS. |
| 16 | Pergunte se a criança tomou outros medicamentos nas últimas 4 semanas. |
| 17 | Se tiver tomado SP ou AQ (para o paludismo) ou Bactrim/Cotrimoxazol (para uma infeção), a criança não deve receber o medicamento de QPS desse dia. |
| 18 | Quando tiver a certeza de que a criança é elegível, poderá prosseguir à administração do medicamento. |
| 19 | Selecione o blíster adequado à idade da criança. |
| 20 | pouse o blíster numa superfície ou mesa. |
| 21 | Explique à responsável pela criança que deverá administrar os DOIS primeiros comprimidos, à criança |
| 22 | Peça à responsável pela criança que acrescente lentamente uma pequena quantidade de água até cobrir os comprimidos e que misture lentamente. |
| 23 | Certifique-se de que a criança está bem acordada e sentada direita. Garanta que a criança não tem a cabeça demasiado inclinada para trás.  Peça à responsável que dê lentamente o tratamento à criança, garantindo que esta engole todo o conteúdo da colher.  Aguarde alguns minutos para se certificar de que a criança fica bem. |
| 24 | Informe a responsável de que, se a criança vomitar TODO o tratamento nos 30 minutos seguintes, deverá ser administrada uma nova dose de tratamento.  Ela deverá ir ao seu encontro e pedir-lhe que regresse para lhe dar um novo blíster.  Não se esqueça de recuperaro blíster antigo.  Se a criança vomitar novamente, aponte o ocorrido no registo. Não lhe dê um novo tratamento. |
| 25 | Após ter concluído o preenchimento do cartão de QPS, coloque-o ao alcance da responsável pela criança e recue. O cartão QPS é um lembrete para o cuidador e um registro dos tratamentos de seu filho.  Preencha a ficha de contabilização indicando o número de crianças que receberam o tratamento durante a visita. |
| 26 | Explique que o tratamento completo deve ser tomado durante TRÊS dias e que a criança deve tomar UM comprimido no dia seguinte e UM comprimido no dia a seguir a esse |
| 27 | Agradeça à família por ter participado na campanha e recorde as mensagens principais   - Lembre-se da data da próxima visita - Guardar o tratamento num local seco e fora do alcance das crianças - Terminar o blíster   **X** Não dar o medicamento a nenhuma outra pessoa   - Os medicamentos da QPS são seguros e bem tolerados. Os efeitos secundários graves são raros. No entanto, se a criança adoecer após ter tomado os medicamentos da QPS, deve ser levada ao centro de saúde. - O tratamento da QPS dá umaproteção de 1 mês. - A QPS é essencial por um período de 3, 4 ou 5 meses, dependendo da duração da estação das chuvas. - Todos os membros do agregado familiar devem dormir sob uma rede mosquiteira impregnada de longa duração de ação (MILDA). - Peça à responsável pela criança que lave as mãos depois de saírem da casa. |
| 28 | Obrigado por ter assistido a este vídeo.  Agora, é capaz de administrar a QPS com toda a segurança, dando uma proteção vital contra o paludismo às crianças. |

**
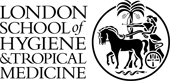

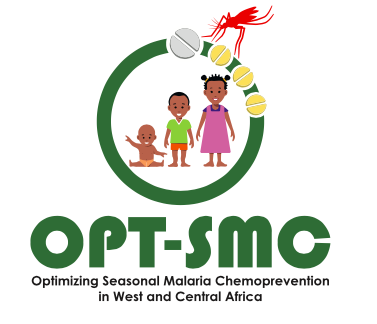
**

**OPT-SMC Training videos**

## English transcript: Delivering Seasonal Malaria Chemoprevention (SMC)

|  | Transcript for Cups |
| --- | --- |
| 1 | After watching this video you will understand how to deliver SMC, to help protect children against malaria during the rainy season. |
| 2 | This year, because of COVID19, all health workers should wear a mask, and avoid close contact with anyone in the household. |
| 3 | When you arrive at a household, explain you and your co-worker have been sent by the district health team to deliver important medication that helps to prevent children from getting sick from malaria. |
| ~~4~~ | Make sure you keep a safe distance from the caregivers and children at all times. |
| 5 | Wash your hands |
| 6 | Now you’re ready to start.    Find a place to sit and ask for a cup, a spoon, and clean drinking water |
| 7 | Set out the things you will need:  ✓ The SMC blister packs  ✓ SMC record cards  ✓ If you are recording SMC treatment digitally, you will need to open the App on your android phone or tablet  If you are not using digital recording you will need:  ✓ the SMC Register  ✓ and the Tally sheet |
| 8 | If this is the first visit of the year, you will need to record details of ALL children under 5 years of age, in the SMC register, or on your device. So ask to see these children.  If a child is under three months, he or she will NOT receive SMC medication during this visit, but they may later in the year, so make sure you register them.  This helps to ensure children are not missed when you return each month. |
| 9 | You will also complete an SMC record card for each eligible child. |
| 10 | Write the child’s name and personal details. You will record the treatment AFTER you have given the SMC medicine. |
| 11 | Before you begin, you need to determine if the child is eligible for SMC. |
| 12 | First, establish how old the child is.  If the caregiver is unsure of the child’s age, ask to see the vaccination card, or ask other members of the household. |
| 13 | If the child is unwell they cannot take SMC now. They must go to the health centre or to a community health worker so the child can be tested. |
| 14 | If they have malaria, they will be given A-C-T treatment. |
| 15 | If the child has had severe side effects from SMC, or from other medicines, in the past, they must not take SMC. |
| 16 | Ask if the child has taken other medicines in the last four weeks. |
| 17 | If they have taken SP, or AQ, (for malaria) or Bactrim or Cotrimoxazole (for an infection), SMC must not be given today. |
| 18 | Once you are confident the child is eligible, you can proceed. |
| 19 | Select the appropriate blister pack for the child’s age. |
| 20 | Place the blister pack on the chair or table |
| 21 | Explain to the caregiver that she will give the first TWO tablets in the pack to the child. |
| 22 | Instruct the caregiver to slowly add a small amount of water to fully cover both tablets at the same time. They may have to softly stir the mixture. |
| 23 | Ensure the child is fully awake and sat upright, avoid tipping the head back too far.    Tell the caregiver to administer slowly, checking the medicine has been swallowed.  Tell them to rinse the cup with a small amount of water and give to the child to swallow again, making sure the cup is now empty.  Wait for a few minutes to ensure all is well with the child. |
| 24 | Tell the caregiver if the child vomits ALL the medicine in the next 30 minutes, they can be redosed. They will need to find you and ask you to come back, so you can give the caregiver a new blister pack. Remember to retrieve the old one, to dispose of later.  If the child vomits again, note the event in the register or in the App on your device. Do not redose. |
| 25 | When you have completed the SMC record card, place it down and step back, so the caregiver can take it. The SMC card is a reminder for the caregiver, and a record of their child’s treatments.  If you are using a tally sheet, , mark to indicate how many children have received SMC during this visit. |
| 26 | Explain this treatment is over THREE days. ONE tablet should be taken tomorrow, and the final tablet to be taken the following day. |
| 27 | Thank the family for taking part in the campaign and remind them of these important messages:  ✓ Remember the date of the next visit  ✓ Keep the pack in a dry place, out of reach of children.  ✓ Finish the blister pack  𝚾 Do not give this medicine to anyone else  ✓ SMC drugs are well tolerated and severe side effects are very rare, but if the child is sick after taking the SMC medicines, take the child to the health facility.  ✓ SMC protects for 1 month, it must be repeated each month, usually for 4 months  ✓ Everyone in your household should sleep under a Long-Lasting Insectide-treated bed net  ✓ Tell the caregiver to wash their hands once you have left the household. |
|  |  |
| 28 | Thank you for watching.    Now you will be able to deliver SMC safely, providing vital malaria protection for children. |
