# Supplementary material for: The use of video job-aids to improve the quality of seasonal malaria chemoprevention delivery
Source: PLOS Digit Health. 2022 Dec 22;1(12):e0000165. doi: 10.1371/journal.pdig.0000165 (PMC9931299; doi:10.1371/journal.pdig.0000165)
Supplement: S3 File — (DOCX) [file pdig.0000165.s003.docx]

**The use of video job-aids to improve the quality of Seasonal Malaria Chemoprevention delivery**

Susana Scott, Bienvenu Salim Camara, Michael Hill, Eugène Kaman Lama, Lansana Barry, Aurore Ogouyemi-Hounto, William Houndjo, Gauthier Tougri, Nombre Yacouba, Dorothy Achu, Marcellin Ateba, Mahamat Saleh Issakha Diar, Keziah L. Malm, Kofi Adomako, Paolo Djata, Wica Da Silva, Idrissa Cissé, Vincent Sanogo, Hadiza Jackou, Nnenna Ogbulafor, Bala M Adu, Jamilu Nikau, Seynabou Gaye, Alioune Badara Gueye, Balla Kandeh, Olimatou Kolley, Tinah Atcha-Oubou, Tchassama Tchadjobo, Kovana Marcel Loua, Andre-Marie Tchouatieu, Ibrahima Mbaye, Maria-Angeles Lima-Parra, Abena Poku-Awuku, Jean Louis Ndiaye, Corinne Merle, Liz Thomas, Paul Milligan

**S3 File: Work sheet for NMCP workshop**

Table of Contents

[A workshop on the training of drug distributors including the use of video 2](#_Toc117766370)

[Section 1: How did your country adapt your SMC training programme as a result of COVID-19 regarding: 2](#_Toc117766371)

[Part a: Materials 2](#_Toc117766372)

[Part b: Training 3](#_Toc117766373)

[Part c: Checking Understanding 3](#_Toc117766374)

[Section 2: The OPT-SMC SMC training video in the context of COVID-19 3](#_Toc117766375)

[Section 3: Using the video in the training for 2021 SMC campaigns 3](#_Toc117766376)


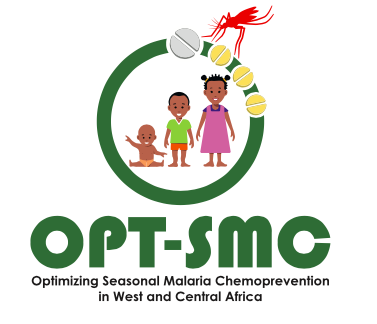


**SMC in the context of Covid – 19**

# A workshop on the training of drug distributors including the use of video

The workshop aims to consider how videos, such as the training video for the delivery of Seasonal Malaria Chemoprevention in the context of COVID-19, can be used to enhance training of drugs distributers with the SMC programme.

The links for the video are here:

**Delivering Seasonal Malaria Chemoprevention (SMC)**

<https://www.youtube.com/watch?v=7HMbQlvFKgQ>

**Administration de la Chimioprevention du Paludisme Saisonner (CPS)**

<https://www.youtube.com/watch?v=mMN81ENZjFM&t=14s>

**Administration de la Chimioprevention du Paludisme Saisonner (CPS) (10 ans)**

<https://www.youtube.com/watch?v=GIBWH0C12BE&list=PLGi4ri37Ak3CCsWZD1uR9G80-RZxE97o2&index=4&t=0s>

Administração da **Quimioprevenção** do **Paludismo Sazonal**  (QPS)

<https://www.youtube.com/watch?v=LvtrxqRHGas&list=PLGi4ri37Ak3CCsWZD1uR9G80-RZxE97o2&index=5&t=2s>

Please use this worksheet to complete your notes on the discussions that will take place during this workshop.

Please answer the following sections whilst we are discussing them during the workshop and afterwards.

## Section 1: How did your country adapt your SMC training programme as a result of COVID-19 regarding:

### Part a: Materials

Review of training tools and materials - because of limited classroom time and need to strengthen training on interpersonal communication – fears and anxieties

- Did you have to change your training materials contents?
- Did you use materials from elsewhere that had been adapted?
- Did you have to develop different or new training materials?
- Did you have to adapt materials because of training the delivery of training?
- Were there any changes that you were not able to make?

*Please add you answers to the above here:*

### Part b: Training

- What did you do differently this year?
- Did you meet face-to-face?
- Did you have to have smaller groups/ meet more often?
- Did you use online training?
- Did you have to use masks?
- Were trainees concerned about risk to themselves

*Please add you answers to the above here:*

### Part c: Checking Understanding

- How did you check that people that understood the (new) processes?
- Who did the checks?
- Were checks done in person on line?
- Did supervisors’ role change?
- What were the main challenges compared to in-person training?

*Please add you answers to the above here:*

## Section 2: The OPT-SMC SMC training video in the context of COVID-19

**English: Delivering Seasonal Malaria Chemoprevention (SMC)**

<https://www.youtube.com/watch?v=7HMbQlvFKgQ>

**French : Administration de la Chimioprevention du Paludisme Saisonner (CPS)**

<https://www.youtube.com/watch?v=mMN81ENZjFM&t=14s>

*While you are watching the video note down any differences in the process between what the video shows and says and what happens in your country. (We’ll come back to these points later).*

## Section 3: Using the video in the training for 2021 SMC campaigns

1. What are the advantages of providing a video to train drugs distributers?
2. What are the limitations?
3. Can you identify any opportunities for using a video to enhance training?
4. How could your country incorporate this, or a similar video, into the training of drug distributers for the 2021 campaign?
5. Using the notes you made while you were watching the video, please tell us about the changes that would make the video more relevant to your context e.g. procedure and protocol, culturally specific issues, language?
6. Is there anything else that would help you to improve the training of drugs distributers?

*Please add you answers to the above here:*
